# The role of K^+^ conductances in regulating membrane excitability in human gastric corpus smooth muscle

**DOI:** 10.1152/ajpgi.00220.2014

**Published:** 2015-01-15

**Authors:** Ji Yeon Lee, Eun-ju Ko, Ki Duck Ahn, Sung Kim, Poong-Lyul Rhee

**Affiliations:** ^1^Department of Medicine,; ^2^Samsung Biomedical Research Institute and; ^3^Department of Surgery, Samsung Medical Center, Sungkyunkwan University School of Medicine, Seoul, Korea

**Keywords:** human stomach, resting membrane potentials, gastrointestinal motility, slow wave potentials, smooth muscle cell

## Abstract

Changes in resting membrane potential (RMP) regulate membrane excitability. K^+^ conductance(s) are one of the main factors in regulating RMP. The functional role of K^+^ conductances has not been studied the in human gastric corpus smooth muscles (HGCS). To examine the role of K^+^ channels in regulation of RMP in HGCS we employed microelectrode recordings, patch-clamp, and molecular approaches. Tetraethylammonium and charybdotoxin did not affect the RMP, suggesting that BK channels are not involved in regulating RMP. Apamin, a selective small conductance Ca^2+^-activated K^+^ channel (SK) blocker, did not show a significant effect on the membrane excitability. 4-Aminopyridine, a Kv channel blocker, caused depolarization and increased the duration of slow wave potentials. 4-Aminopyridine also inhibited a delayed rectifying K^+^ current in isolated smooth muscle cells. End-product RT-PCR gel detected Kv1.2 and Kv1.5 in human gastric corpus muscles. Glibenclamide, an ATP-sensitive K^+^ channel (K_ATP_) blocker, did not induce depolarization, but nicorandil, a K_ATP_ opener, hyperpolarized HGCS, suggesting that K_ATP_ are expressed but not basally activated. Kir6.2 transcript, a pore-forming subunit of K_ATP_ was expressed in HGCS. A low concentration of Ba^2+^, a Kir blocker, induced strong depolarization. Interestingly, Ba^2+^-sensitive currents were minimally expressed in isolated smooth muscle cells under whole-cell patch configuration. *KCNJ2* (Kir2.1) transcript was expressed in HGCS. Unique K^+^ conductances regulate the RMP in HGCS. Delayed and inwardly rectifying K^+^ channels are the main candidates in regulating membrane excitability in HGCS. With the development of cell dispersion techniques of interstitial cells, the cell-specific functional significance will require further analysis.

in gastric corpus and antrum, many studies have focused on the mechanisms of spontaneous electrical activity (4, 7, 9, 10, 25, 29, 33). Interstitial cells of Cajal (ICC) have been shown to generate or initiate spontaneous electrical activity in gastric smooth muscle. Changes in the resting membrane potentials (RMP) of the gastric smooth muscle can affect excitability of slow wave potentials (SWP). For instance, slow wave depolarization drives the changes in rhythmic contractility in gastric smooth muscle. The RMP can mainly be decided by the expression of K^+^ channels, although other inward conductance can affect the RMP (3, 19). Voltage-dependent and -independent K^+^ channels can be involved in setting RMP. Large-conductance Ca^2+^-activated K^+^ channels (BK) and voltage-dependent delayed rectifying K^+^ channels (Kv) are common candidates for voltage-dependent K^+^ channels. Voltage-independent K^+^ channels such as small-conductance Ca^2+^-activated K^+^ channels (SK), ATP-sensitive K^+^ channels (K_ATP_), and inwardly rectifying K^+^ channels (Kir) may be involved in regulating RMP (19).

It has been shown that BK channel blockers [tetraethylammonium chloride (TEA) or charybdotoxin (ChTX)] did not affect the RMP in colonic smooth muscles. 4-Aminopyridine (4-AP, a Kv channel blocker) induces depolarization in colonic smooth muscle, suggesting that activation or inhibition of Kv channels can affect the changes in RMP (17, 18). Apamin (a specific SK channel blocker) and glibenclamide (GBC, a K_ATP_ channel blocker) also depolarize colonic smooth muscle (16, 21). These data suggested that ongoing activities of SK and K_ATP_ channels are important conductances in regulating RMP. Low concentrations of Ba^2+^ (μM range) inhibit Kir channels. Ba^2+^ also induces depolarization in colonic smooth muscle (6).

Although electrical activity with use of intracellular microelectrodes was reported in human gastric smooth muscles (4, 7, 25), there is no report about the role of K^+^ conductances on membrane excitability in human gastric corpus smooth muscle (HGCS). Understanding the role of K^+^ channel in HGCS is crucial to explain the spontaneous electrical rhythmicity. Unfortunately, isolation of interstitial cells for patch experiment in HGCS is technically limited. Therefore, we focused on K^+^ channel expression in smooth muscle using microelectrode recordings, patch-clamp, and molecular analysis.

## MATERIALS AND METHODS

### 

#### Human gastric corpus tissue preparation.

All studies were approved by the Institutional Review Board of the Samsung Medical Center (no. 2010-09-015). A nonpathological segment of human gastric corpus tissues was used in this study obtained from gastrectomy patients (*n* = 36, average age 64) of either sex at Samsung Medical Center. The mucosa was removed from the sample by sharp dissection. Thin strips of tissues were cut from the sample by use of parallel scalpel blades mounted on a scalpel handle. The final strips cut parallel to the longitudinal muscle fibers measured 10 mm (or less) in length. Tissues were pinned to Sylgard elastomer-coated Corning dish and placed in a recording chamber. The tissues were incubated at 37 ± 0.5°C with oxygenated Krebs-Ringer bicarbonate solution (KRB; see below).

#### Intracellular microelectrode recordings.

After 1-h incubation in the recording chamber, microelectrode impalements of circular smooth muscle cells were made with glass microelectrodes having resistances of 80–120 MΩ with 3 M KCl. Transmembrane potentials were recorded with an Axon Instruments high-impedance microelectrode amplifier and data were recorded onto a PC running Axo Scope 10.0 data acquisition software (Molecular Devices, Union City, CA). Data were analyzed by Clampfit 10.0 (Molecular Devices). All experiments were performed in the presence of tetrodotoxin (TTX, 1 μM).

#### Smooth muscle cell preparation.

Freshly dispersed smooth muscle cells were prepared from HGCS strips with use of Ca^2+^-free Hanks' solution containing (in mmol/l) 125 NaCl, 5.36 KCl, 15.5 NaOH, 0.336 Na_2_HPO_4_, 0.44 KH_2_PO_4_, 10 glucose, 2.9 sucrose, and 11 HEPES, adjusted to pH 7.4 with Tris. Dissected muscle strips were incubated for 40–50 min at 37°C in a Ca^2+^-free solution (2 ml) containing collagenase (4 mg/ml, Worthington Biochemical, Lakewood, NJ), trypsin inhibitor (8 mg/ml), fatty acid-free bovine serum albumin (8 mg/ml), papain (2 mg/ml), and l-dithiothreitol (0.3 mg/ml, Sigma-Aldrich, St. Louis, MO). Tissue pieces were washed with Ca^2+^-free solution and then gently agitated to create a cell suspension. Dispersed smooth muscle cells were stored at 4°C in Ca^2+^-free solution. Cell suspensions were placed on the bottom of a 300-μl chamber mounted on an inverted microscope and allowed to adhere to the bottom of the chamber for 5 min before recording.

#### Patch-clamp experiments.

The whole-cell voltage-clamp technique was used to record membrane currents from dissociated HGCS cells. Currents were amplified with an Axopatch 200B (Axon Instruments, Foster City, CA). Data were digitized with 16-bit analog to digital converter (Digidata 1322A, Axon Instruments). Data were stored directly and digitized online by use of pClamp software (version 9.0, Axon Instruments). The data were sampled at 5 KHz with low-pass filter at 2 KHz by use of an eight-pole Bessel filter. Experiments were performed at room temperature (between 22 and 25°C).

#### Solutions and drugs.

In intracellular microelectrode recordings, the tissue chamber housing muscles was constantly perfused with oxygenated KRB solution of the following composition (in mmol/l): 120.4 NaCl, 5.9 KCl, 1.2 MgCl_2_, 15.5 NaHCO_3_, 1.2 NaH_2_PO_4_, 11.5 glucose, 2.5 CaCl_2_. The pH of the KRB was 7.3–7.4 when bubbled with 97% O_2_-3% CO_2_ at 37.0 ± 0.5°C. To measure net outward currents, smooth muscle cells were bathed in a Ca^2+^-containing physiological salt solution (CaPSS) containing (in mmol/l) 135 NaCl, 5 KCl, 2 CaCl_2_, 1.2 MgCl_2_, 10 glucose, 10 HEPES adjusted to pH 7.4 with Tris. Cells were dialyzed with solution containing (in mmol/l) 135 KCl, 5 EGTA, 2 CaCl_2_, 0.1 Na_2_GTP, 3 MgATP, 10 glucose, 2.5 creatine phosphate disodium, and 10 HEPES and was adjusted to pH 7.2 with Tris. Free Ca^2+^ concentration was 100 nM. In a Ca^2+^-free containing physiological salt solution (MnPSS), external CaCl_2_ was replaced with an equimolar MnCl_2_. To increase the driving force for K^+^ influx in human gastric smooth muscle cells, cells were perfuse in high K^+^-containing solution (HK, 135 mmol/l Na^+^ was replaced with equimolar K^+^) and the pipette solution contained (in mmol/l): 135 KCl, 10 BAPTA, 0.1 Na_2_GTP, 3 MgATP, 10 glucose, 2.5 creatine phosphate disodium, and 10 HEPES and was adjusted to pH 7.2 with Tris. TEA, 4-AP, nicorandil (NCD), GBC, BaCl_2_, E-4031, ketoconazole, and ChTX were purchased from Sigma-Aldrich. TTX was purchased from Alomone Labs (Jerusalem, Israel), and apamin was purchased from Tocris Bioscience (Bristol, UK).

#### Molecular studies.

Total RNA isolation, cDNA preparation, and amplification of HGCS strips (*n* = 3, circular muscle layer) were performed as previously reported (16). Briefly, RNA was prepared by using a SNAP Total RNA isolation kit (Invitrogen, San Diego, CA) per the manufacturer's instructions. RNA was treated with RNase-free DNase I (2 units) at 37°C (New England Biolabs) prior to cDNA preparation. First strand cDNA was synthesized from each RNA by using Superscript II Reverse Transcriptase with 500 μg/μl of oligo(dT) primer cDNA. To investigate the transcriptional expression of K^+^ channels, the following PCR primers designed against human sequences were used (GenBank accession number is given in parenthesis for the reference nucleotide sequence used and common name): *KCNA1* (NM_000217, Kv1.1), *KCNA2* (NM_004974, Kv1.2), *KCNA3* (NM_002232, Kv1.3), *KCNA4* (NM_002233, Kv1.4), *KCNA5* (NM_002234, Kv1.5), *KCNA6* (NM_002235, Kv1.6), *KCNA7* (NM_031886, Kv1.7), *KCNB1* (NM_004975, Kv2.1), *KCNB2* (NM_004770, Kv2.2), *KCNV1* (NM_014379, Kv2.3), *KCND1* (NM_004979, Kv4.1), *KCND2* (NM_012281, Kv4.2), *KCND3* (NM_004980, Kv4.3), *KCNN1* (NM_002248, SK1), *KCNN2* (NM_021614, SK2), *KCNN3* (NM_002249, SK3), *KCNN4* (NM_002250, SK4), *KCNJ2* (NM_000891, Kir2.1), *KCNJ3* (NM_002239, Kir3.1), *KCNJ5* (NM_000890, Kir3.4), *KCNJ6* (NM_002240, Kir3.2), *KCNJ8* (NM_004982, Kir6.1), *KCNJ9* (NM_004983, Kir3.3), and *KCNJ11* (NM_000525, Kir6.2).

#### Statistical analysis.

Data were expressed as means ± SE. The paired Student's *t*-test was used where appropriate to evaluate differences in the data. *P* values less than 0.05 were taken as statistically significant differences. The number of recordings from muscle strips in microelectrode experiments and from cells in patch-clamp experiments is indicated by *n*.

## RESULTS

### 

#### Role of BK and Kv conductances in regulating the membrane excitability of HGCS.

**Fig. 1.**
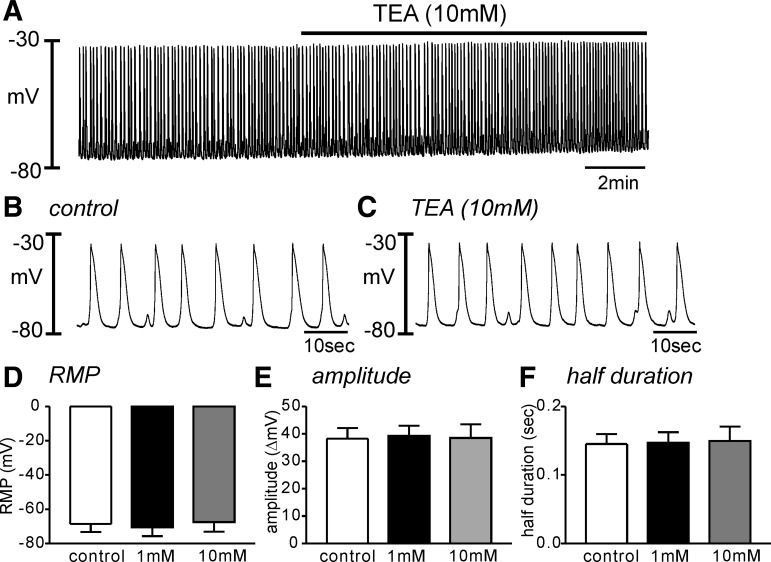
Effect of tetraethylammonium (TEA) on the slow wave potential in human gastric corpus smooth muscles (HGCS). *A*: a representative trace shows that high concentration of TEA (10 mM) had no effect on the slow wave potentials in HGCS. *B* and *C*: expanded time scale from *A* in control (*B*) and TEA presence (*C*), respectively. *D*–*F*: summarized data show no significant changes in the resting membrane potential (RMP, *D*), slow wave amplitude (*E*), and half-duration of slow wave potentials (*F*) from 5 samples in each concentration of TEA.

The role of K^+^ conductance(s) on membrane excitability of HGCS has not been reported. We tested the effect of TEA (1–10 mM) to examine the role of BK and Kv on electrical events in HGCS. The RMP in control tissue was −69 ± 5 mV. TEA application did not affect the RMP (−70 ± 5 mV in 1 mM *n* = 5, Fig. 1, *A*–*D*). High concentration of TEA (10 mM) did not affect the amplitude and duration of SWP (Fig. 1, *E* and *F*). Since high concentration of TEA can block Kv channels, we also tested the effect of ChTX, a specific blocker of BK and intermediate-conductance Ca^2+^-activated K^+^ channels, on the RMP. ChTX also did not show any significant effects on RMP (*n* = 4, data not shown). These data suggest that BK and TEA-sensitive Kv channels have a negligible role on the SWP.

**Fig. 2.**
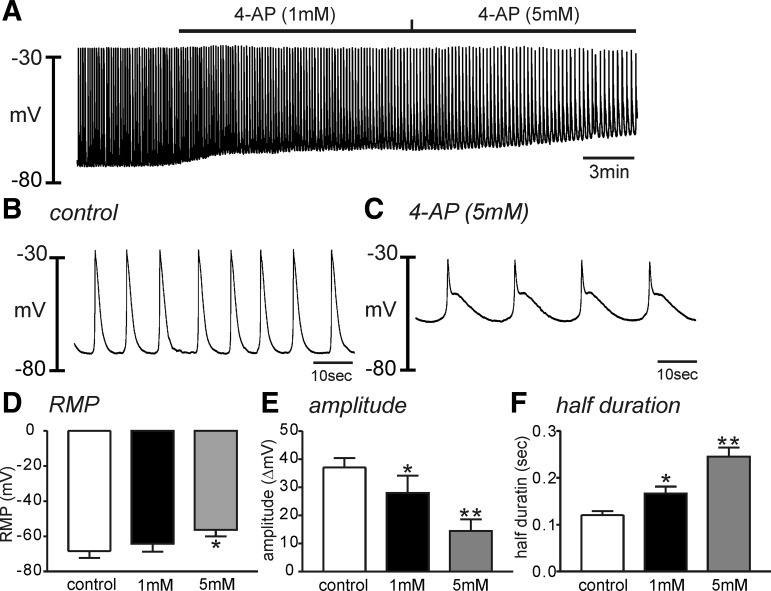
Effect of 4-aminopyridine (4-AP) on the slow wave potential in HGCS. *A*: a representative trace showed that low (1 mM) and high (5 mM) concentration of 4-AP depolarized HGCS with decreased slow wave amplitude and frequency. *B* and *C*: expanded time scale from *A* in control (*B*) and 4-AP (5 mM) presence (*C*), respectively. *D*–*F*: summarized data showed significant changes in the resting membrane potential (RMP, *D*), slow wave amplitude (*E*), and half duration of slow wave potentials (*F*) from 5 samples in each concentration of 4-AP. *P < 0.05; **P < 0.01.

To examine the role of Kv channels on RMP, we tested the effects of 4-AP on electrical events in HGCS. 4-AP (1 mM) induced depolarization but not significantly (e.g., from −68 ± 4 mV to −64 ± 4 mV, *P* = 0.09, *n* = 5). Higher concentration of 4-AP (5 mM) depolarized HGCS, significantly (−56 ± 4 mV, *P* < 0.05 compared with control, *n* = 5, Fig. 2, *A*–*D*). 4-AP significantly decreased the amplitude of SWP (Fig. 2, *A*–*C* and *E*) and increased the half-duration of SWP from 0.12 ± 0.01 to 0.25 ± 0.02 s (*P* < 0.01, *n* = 5). These data suggest that 4-AP-sensitive Kv channels are involved in regulating RMP and membrane repolarization in HGCS.

#### Role of K_ATP_ conductance in regulating the membrane excitability of HGCS.

**Fig. 3.**
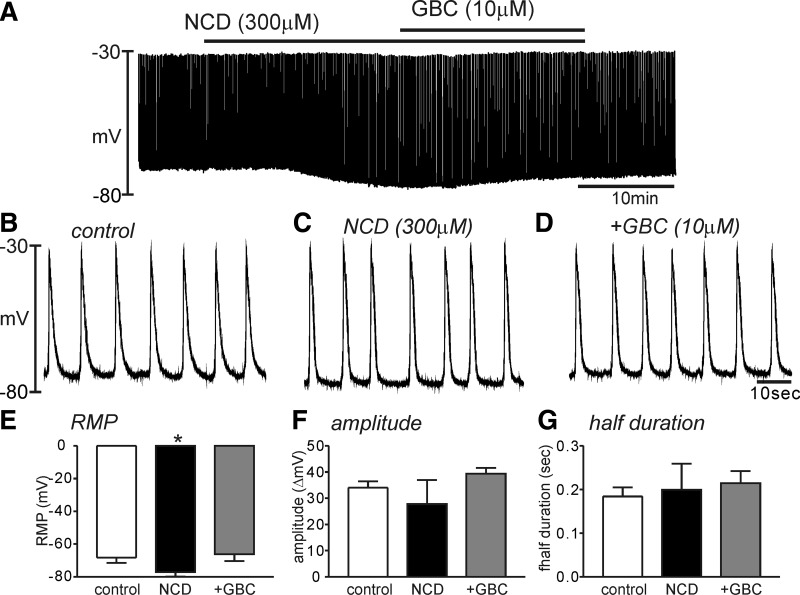
Effect of nicorandil (NCD) and glibenclamide (GBC) on the slow wave potential in HGCS. *A*: a representative trace showed that NCD (300 μM) induced hyperpolarization and continuous application of GBC (10 μM) abolished the NCD effect. *B*–*D*: expanded time scale from *A* in control (*B*), NCD presence (*C*), and both NCD and GBC presence (*D*), respectively. *E*–*G*: summarized data showed significant changes in the resting membrane potential (RMP, *E*) but no significance on the slow wave amplitude (*F*) and slow wave duration (*G*) from 6 samples in the presence of NCD and NCD+GBC. *P < 0.05.

It has been suggested that basally activated K_ATP_ can be involved in regulating RMP (16). We tested the effect of a K_ATP_ blocker, GBC, on SWP in HGCS. GBC (10 μM) itself did not show any significant effect on the RMP (control; −67 ± 3 mV vs GBC; −66 ± 3 mV, *n* = 6). However, the K_ATP_ activator (NCD, 300 μM) hyperpolarized HGCS from −67 ± 2 mV to −77 ± 2 mV (*P* < 0.01, *n* = 6). Interestingly, NCD did not show significant effects on the half-duration of SWP (*n* = 5 Fig. 3, *A*, *B*, and *G*). Some tissues (2 of 5 samples) completely stop the slow wave. Thus the amplitude of SWP by NCD revealed the big standard error and showed no statistical significance (Fig. 3*F*). NCD-induced hyperpolarization was completely recovered to resting potential by GBC (10 μM) (*n* = 6, Fig. 3, *A*–*G*). The pretreatment of GBC also completely abolished the effect of NCD (data not shown).

#### Role of SK conductance in regulating the membrane excitability of HGCS.

**Fig. 4.**
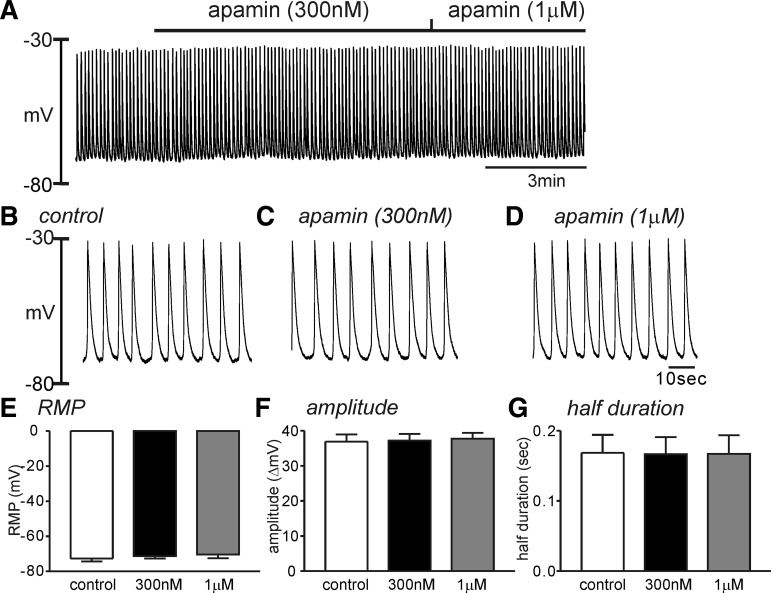
Effect of apamin on the slow wave potential in HGCS. *A*: a representative trace showed that apamin (300 nM and 1 μM) had no effect on the slow wave potentials in HGCS. *B*–*D*: expanded time scale from *A* in control (*B*), apamin (300 nM) presence (*C*), and apamin (1 μM) presence (*D*), respectively. *E*–*G*: summarized data showed no significant changes in the resting membrane potential (RMP, *E*) but no significance on the slow wave amplitude (*F*) and half-duration of slow wave potentials (*G*) from 5 samples in each concentration.

Apamin, a SK channel blocker, is known to induce depolarization in various regions of gastrointestinal smooth muscles (19). However, it has also been reported that apamin had no effect on the RMP in guinea pig antrum (19). The effect of apamin in HGCS has not been studied. Apamin (300 nM–1 μM, *n* = 5 each concentration) did not affect the RMP, amplitude, and frequency of SWP in HGCS (Fig. 4), suggesting that the basal activation of SK channels in regulating RMP is negligible.

#### Role of Kir conductance in regulating the membrane excitability of HGCS.

**Fig. 5.**
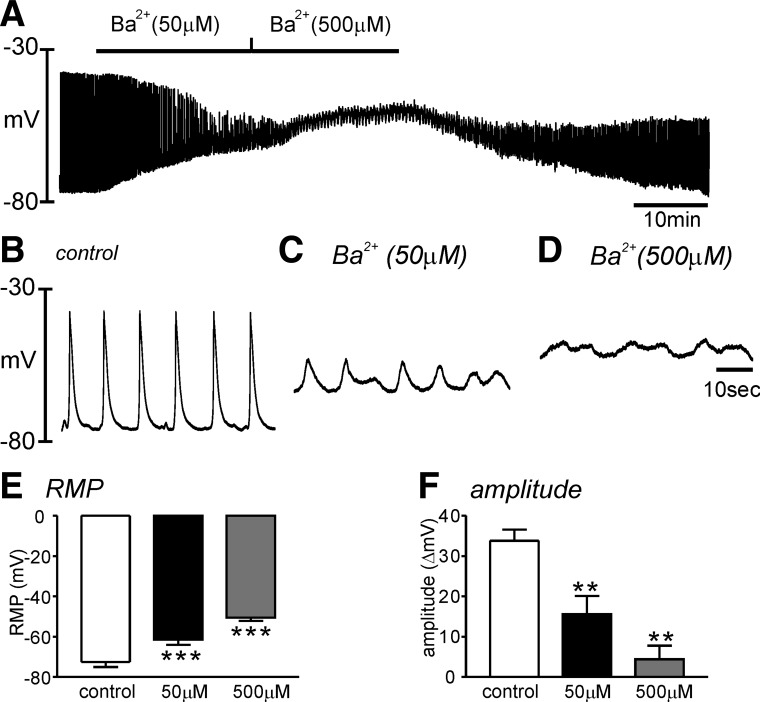
Effect of Ba^2+^ on the slow wave potential in HGCS. *A*: a representative trace showed that Ba^2+^ (50–500 μM) significantly depolarized HGCS. *B*–*D*: expanded time scale from *A* in control (*B*), low concentration of Ba^2+^ (50 μM) (*C*), and high concentration of Ba^2+^ (500 μM) (*D*), respectively. *E* and *F*: summarized data showed significant changes in the resting membrane potential (RMP, *E*) and the slow wave amplitude (*F*) from 5 samples in each concentration. **P < 0.01; ***P < 0.001.

Kir can be blocked by low concentration of Ba^2+^ (6, 19). Bath application of Ba^2+^ (50 μM) depolarized HGCS from −72 ± 3 mV to −61 ± 3 mV (*P* < 0.001, *n* = 5, Fig. 5, *A*–*C*). A higher concentration Ba^2+^ (500 μM) induced strong depolarization of HGCS to −51 ± 2 mV (*P* < 0.001, *n* = 5, Fig. 5, *A* and *E*). Ba^2+^ also significantly decreased the amplitude (Fig. 5*F*) of SWP. Ba^2+^ (500 μM, *n* = 3 of 5 experiments) completely abolished SWP with strong depolarization. Therefore, we could not analyze the effect of Ba^2+^ on the duration of SWP. These data suggest that Kir channels are involved in regulating RMP of HGCS.

#### Role of HERG conductance in regulating membrane excitability in HGCS.

**Fig. 6.**
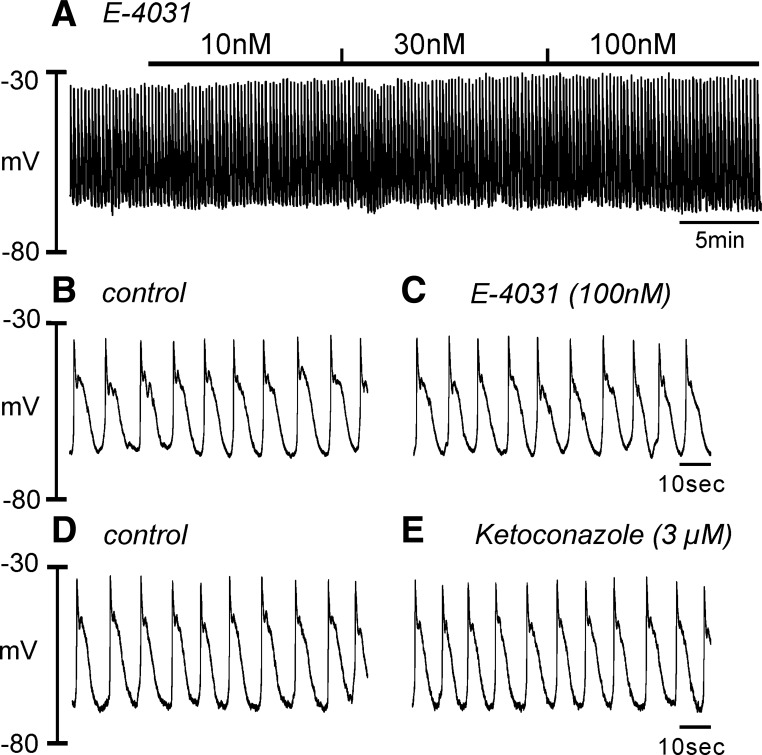
Effect of E-4031 and ketoconazole on the slow wave potential in HGCS. *A*: a representative trace showed that E-4031 (10 nM–1 μM) had no effect on the slow wave potentials in HGCS. *B* and *C*: expanded time scale from *A* in control (*B*) and after E-4031 (100 nM) (*C*). *D* and *E*: representative traces showed no effect of ketoconazole (3 μM) before (*D*) and after (*E*).

The functional role of HERG channels in RMP regulation was also tested. The compound E-4031 (10 nM–1 μM), a HERG channel blocker, did not affect the RMP, amplitude, and half-duration of slow wave (*n* = 4, Fig. 6, *A*–*C*). Ketoconazole (3 μM, *n* = 4), another HERG channel blocker, also did not show significant changes in RMP (Fig. 6, *D* and *E*). However, a high concentration (30 μM) of ketoconazole depolarized HGCS. This might be due to nonspecific blocking of ketoconazole on other K^+^ conductances.

#### Current isolation of Kv and Kir conductance in isolated smooth muscle cells of HGCS.

**Fig. 7.**
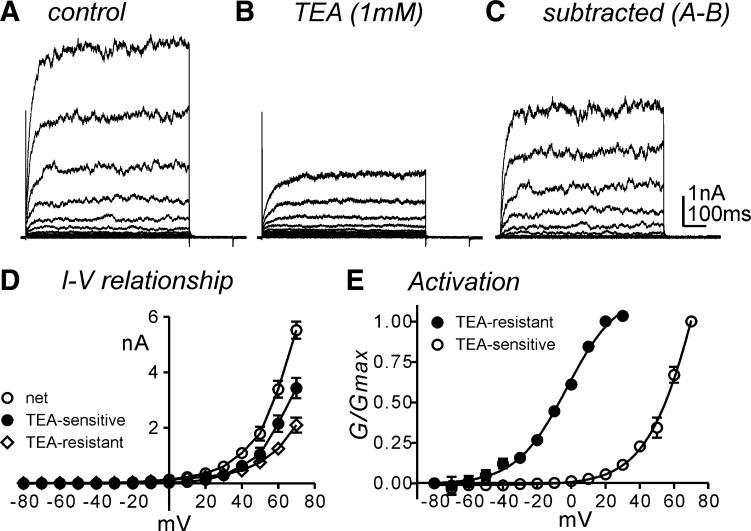
Characteristics of the outward currents in isolated human gastric corpus smooth muscle cells. *A*: a representative trace showed outward currents during step depolarization up to +70 mV in 10 mV increment from a holding potential of −80 mV. Cells were exposed to Ca^2+^-containing physiological solution (CaPSS) externally and dialyzed with K^+^-rich solution (100 nM intracellular Ca^2+^ concentration) internally. *B*: TEA (1 mM) decreased outward currents and remained the delayed rectifying K^+^ currents (TEA-resistant). *C*: subtracted currents from *A* to *B* showed TEA-sensitive currents (mainly BK). *D*: summarized current-voltage (*I*–*V*) relationship of the net outward (control, ○) and BK (TEA-sensitive, ●) and delayed rectifying K^+^ (TEA-resistant, ◇) currents. *E*: voltage-dependent activation from TEA-resistant (●) and TEA-sensitive (○) currents. Boltzmann equation was applied to calculate half activation in normalized with maximal conductance (*G*/*G*_*max*_).

To examine the characteristics of net outward currents, isolated smooth muscle cells were exposed to CaPSS (see methods) and dialyzed with K^+^-rich solution (intracellular Ca^2+^ concentration, [Ca^2+^]_i_; 100 nM). Cells were held at −80 mV and depolarized to +70 mV with 10-mV increments (Fig. 7, *A* and *D*). TEA (1 mM, *n* = 5) or ChTx (200 nM, *n* = 3 data not shown) to inhibit BK channels decreased the outward currents and remained TEA-resistant currents (Fig. 7, *B* and *D*). TEA-resistant currents showed a classical property of delayed rectifying K^+^ currents without voltage-dependent inactivation within a 700-ms depolarization period. TEA-sensitive currents (mainly BK currents, Fig. 7, *C* and *D*) were constructed by subtraction from control (Fig. 7*A*) to TEA-resistant (Fig. 7*B*) currents. TEA-sensitive and TEA-resistant currents showed voltage-dependence with a half activation voltage of +73 ± 3 mV and −3 ± 0.7 mV, respectively (Fig. 7*E*).

**Fig. 8.**
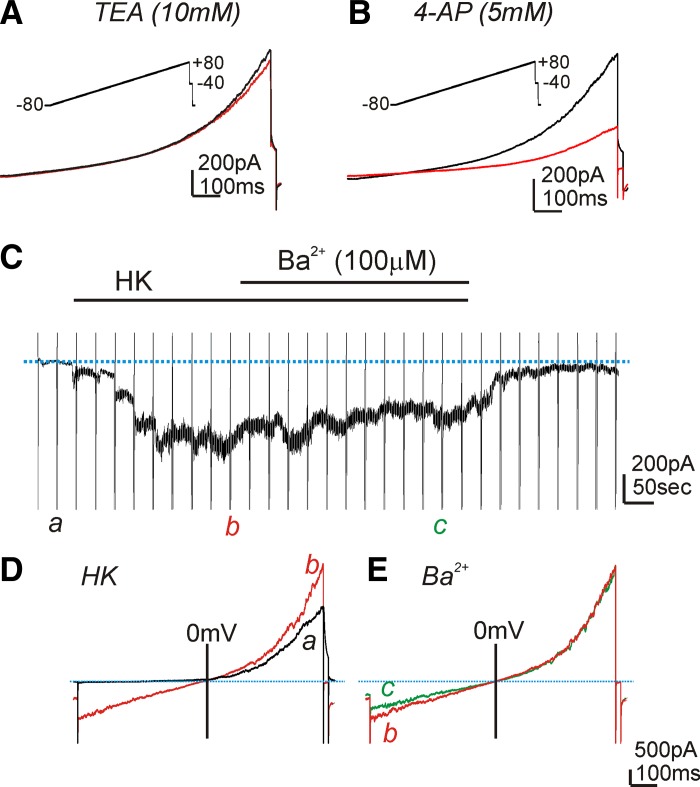
Effect of TEA, 4-AP, and Ba^2+^ on the outward currents in isolated HGCS cells. *A* and *B*: a representative trace showed outward currents during ramp depolarization (from −80 to +80 mV; see *inset*) from a holding potential of −80 mV. TEA (10 mM) did not show significant effect on evoked outward currents (*A*). However, 4-AP (5 mM) decreased the outward currents (*B*). *C*: a representative trace showed the effect of Ba^2+^ (100 μM) after replacement with K^+^-rich solution (HK; see methods) at −80 mV. Vertical lines denote ramp depolarization and blue dotted line denotes the holding current at −80 mV. *D*: *a* (black line) and *b* (red line) are evoked currents during ramp depolarization from *C* in control and HK presence, respectively. *E*: bath application of Ba^2+^ (100 μM, *c*: green line) under external K^+^-rich solution (*b*: red line) at a holding potential of −80 mV did not reveal significant effect on ramp-evoked currents.

Since the most prominent findings by microelectrode recordings in HGCS are 4-AP and Ba^2+^ sensitive, we performed patch-clamp experiments to characterize the 4-AP and Ba^2+^-sensitive currents from isolated smooth muscle cells of the HGCS. Whole-cell voltage-clamp techniques were employed. The external solution was MnPSS (see methods) with dialyzed K^+^-rich solution (10 mM BAPTA to prevent the contamination of Ca^2+^-activated K^+^ currents; see methods). Ramp depolarization to +80 mV from a holding potential of −80 mV was applied (see *insets* in Fig. 8, *A* and *B*). A high concentration of TEA (10 mM) had no effect on the evoked currents (Fig. 8*A*, *n* = 5), but 4-AP (5 mM) inhibited outward currents from 158 ± 12 pA to 68 ± 6 pA at 0 mV (Fig. 7*B*, *P* < 0.01, *n* = 6). These data suggest that HGCS smooth muscle cells express 4-AP-sensitive Kv currents.

To examine the functional expression of Kir channels in isolated smooth muscle cells, we changed the external solution from MnPSS to HK (140 mM; see methods) to increase the driving force of K^+^ at the negative potentials. The replacement of the external solution from MnPSS to HK activated inward currents (−335 ± 36 pA) at a holding potential of −80 mV (see Fig. 8*C*). Ramp depolarization evoked huge inward currents in HK compared with MnPSS at the negative potentials (Fig. 8*D*). The addition of Ba^2+^ (100 μM) minimally inhibited the inward currents (−305 ± 46 pA at −80 mV, Fig. 8, *C* and *E*, *n* = 5). These data suggest that Kir conductance of HGCS smooth muscle cells may not have a functional role in setting RMP.

Taken together, the effects of 4-AP on membrane potentials could be due to Kv channels in smooth muscle cells but Ba^2+^-induced depolarization in tissue is not due to expression of Kir channels in smooth muscle cells.

#### Molecular expression of K channels in HGCS.

**Fig. 9.**
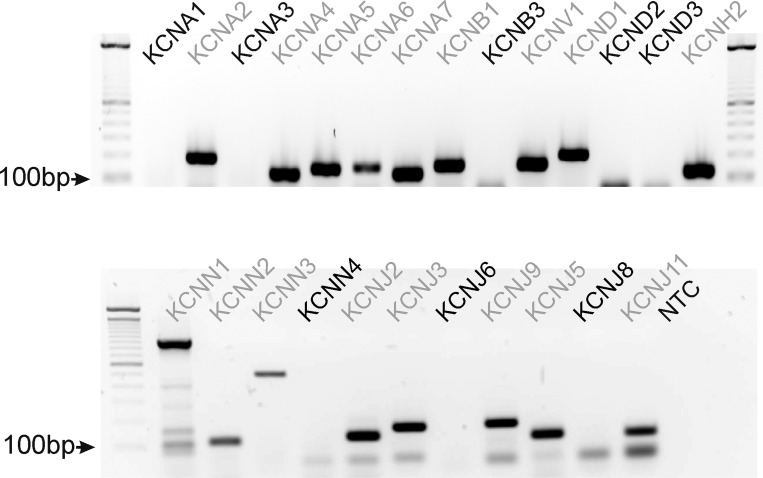
Transcriptional expression of K^+^ channels in HGCS. Representative agarose end-point gel of RT-PCR products revealed K^+^ channels expression in HGCS. Light gray font denotes the detected isoforms of tested K^+^ channel transcripts.

To examine transcriptional expression of K^+^ channels in HGCS, we performed RT-PCR analysis (see Table 1 for the specific primers). *KCNNA2*, *4*, *5*, *7*, *KBNB1*, and *KCNV1*, Kv molecular candidates, were detected in HGCS. *KCNJ2* but not *KCNJ1*, K_ATP_ candidates, was expressed. All *KCNN1–3*, SK channel candidates, but not *KCNN4*, an intermediate conductance Ca^2+^-activated K^+^ channel candidate, were detected. *KCNJ2*, *3*, *5*, *9*, *and KCNH2* were also expressed in HGCS (Fig. 9).

**Table 1.** Primer sequence of tested genes

Gene Name	Primer Sequence	Gene Name	Primer Sequence
KNCA1	TGATGTCTGGGGAGAACGTG	KCNH2	CACACATGGACTCACGCATC
	GCAGCCCGGAGATGTTGAT		TAGAGCGCCGTCACATACTT
KCNA2	CATGAGAGAATTGGGCCTCC	KCNN1	TCGGGGAAACCCTCAAATGT
	CCCCCAATGGTAGTCGGAAC		GCCAATGGAGAGGAAGGTGA
KCNA3	GCGACGAGAAGGACTACCC	KCNN2	TCGAAAAGCGCAAGCGGCTC
	GCTAGGACAAGCGAAGAACC		AGCGACGCCTTGTCGTAGGC
KCNA4	TACCTCCCATGACCCTCAGA	KCNN3	ACCAAGCGGATCAAGAATGC
	GGCATCAGGTCAGAGCAATG		ATGGGGCTATCGGAGATTGG
KCNA5	TGTTCGCGGACGAGATACG	KCNN4	GCCTTGAGACGCCGAAAGCG
	ACTCGAAGATAAGCCACACCT		CAGGTAGAGCGCCCACGAGC
KCNA6	TTTGCCTGGAGACCTTACCC	KCNJ2	GCTCATGTGTAGTGTGCGAG
	AGGGGTGATGCCATGATGAA		GAGAATGCCAGGATGCCAAG
KCNA7	CCGGACACTCTGCTAGGGG	KCNJ3	GCTACCTCTCGGACCTCTTC
	GACTGGTAGTAGTAGAGCACGG		TAGTTACCGACGTGGGCTTT
KCNB1	CCAGTGGTCAGGAAAAGTGC	KCNJ6	GACAGAATCCATGACTAACGTCC
	ACAGCTCTCCTCTTTGGACC		TCCGTCTTTCCTCACGTACC
KCNB2	CATCAGTGGCTGCAAAGATCC	KCNJ9	ATGGACTTTTGGGGGTTGGA
	GTGCTAATTGGCGGTTGTCA		TGGTTTGGGGCTGTATCTGT
KCNV1	ACAGCGTGAAGCCCTAAAGA	KCNJ5	GCACATGCATAGTGGCATCA
	ACCAGAAATCATCTCCCCCG		TCTTGGGCCAAAAAGCACTC
KCND1	CCTGTGCCAGTCATTGTGTC	KCNJ8	TGGCTGCTCTTCGCTATCAT
	TTTGCCAATCGGATCCTTGC		AGCAGAAGTGAAAGACCTGAC
KCND2	GTTAGCAAATCCGGCTCCAG	KCNJ11	TCCAAGAAAGGCAACTGCAAC
	TGCAACTTCCATGCAGCTTT		AAGATGAGCAATGTGTGTGGC
KCND3	ACCTCCACCATCAAGAACCA		
	GCTGGCAGGTTAGAATTGGG		

## DISCUSSION

The generation of slow waves by ICC is well established (25, 27, 28, 30). The RMP in gastrointestinal muscle can be regulated by electrically coupled conductances in SIP (Smooth muscle cells, ICC and PDGFRà^+^ cells) syncytium (smooth muscle cells; ICC and PDGFRα^+^ cells) (19, 28). A small depolarization from RMP activates voltage-dependent Ca^2+^ channels and affects contractility. Although the electrical activity was recorded in the human stomach (4, 7, 25), the role of basally activated K^+^ conductances to regulate RMP in HGCS has not been studied. In the present study, we elucidate the role of K^+^ conductances, which regulate RMP in HGCS. This is only the first study to investigate the role of K^+^ channels in the electrical activity of human gastric muscles.

### 

#### Role of Kv channels in HGCS.

Smooth muscle excitability in HGCS can be regulated by enteric nervous system (31). We used TTX, a Na^+^ channel blocker, throughout experiments to exclude neural influences. It has been suggested that TTX augmented spontaneous colonic contractility through blocking tonic inhibitory enteric motor influence (14). However, in HGCS, TTX did not display any significant effects on spontaneous SWP. These data suggest that inhibitory neurotransmitters are not dominating factors in ongoing membrane excitability of HGCS. Basally activated BK channels can affect the setting of the RMP. In the present study, BK channel blockers, ChTX, or a low concentration of TEA (1 mM) did not affect the RMP or SWP patterns in HGCS. Furthermore, in patch-clamp experiments, TEA- or ChTX-sensitive currents were activated at the positive potentials in intracellular 100 nM free [Ca^2+^]_i_ condition. These data suggest that BK channels might have a negligible role on membrane excitability in HGCS.

Kv channels are known to regulate membrane excitability in colonic smooth muscles (19). Kv channels can be blocked by high concentrations of TEA (up to 10 mM) and 4-AP (5 mM). High concentration of TEA (10 mM) did not affect SWP but 4-AP induced depolarization with an increase in the duration of SWP of HGCS. These data suggest that 4-AP-sensitive Kv channels might be expressed in HGCS. The transcriptional expression of *KCNA2* (Kv1.2), *KCNA4* (Kv1.4), *KCNA5* (Kv1.5), *KCNA7* (Kv1.7), *KCNB1* (Kv2.1), and *KCND1* (Kv4.1) was detected in HGCS. *KCNA4* and *KCND1* revealed the fast inactivation during depolarization (24, 32). In the present study, low concentration of TEA (1 mM) inhibited BK channels, and there remained Kv (TEA-resistant) currents that showed a negligible inactivation property during 700-ms depolarization. *KCNB1* is very sensitive to TEA (8, 12). Under MnPSS with BAPTA dialysis (see methods) to exclude the contamination of BK currents, TEA (10 mM) did not show a significant effect on Kv currents. In addition, there is no report about the effect of 4-AP or TEA on *KCNA7*. In smooth muscles of many animal models, TEA-sensitive currents are available and have potent impact on the electrical activity. This appears not to be true for human gastric muscles. 4-AP-sensitive currents appear to be important in human muscles both for setting membrane potential and for determining the duration of slow wave events. Thus *KCNA2* and *KCNA5* could be molecular candidates for 4-AP-sensitive Kv channels in smooth muscle cells. Taken together, Kv channels in smooth muscle cells of the HGCS could be the main K^+^ conductances in regulating RMP and SWP.

#### Role of SK and K_ATP_ channels in HGCS.

Apamin is a selective blocker for SK channels (1). SK channels are voltage independent and sensitive to intracellular Ca^2+^. SK channels are expressed in gastrointestinal smooth muscle (15). The effect of apamin on the RMP is not consistent depending on the species and tissues. For instance, colonic smooth muscles are depolarized by apamin (19) but guinea pig antrum is not sensitive to apamin (20). In the present study, we did not find the effect of apamin on the RMP of HGCS, suggesting that SK channels are not basally active. In murine colonic smooth muscle cells, SK2 (*Kcnn2*) is a dominant isoform (15, 26). In recent reports, PDGFRα^+^ cells highly express SK3 (*Kcnn3*) and involves purine-mediated inhibitory junction potentials (11, 22, 34). In HGCS, *KCNN1* (SK1, K_Ca_2.1), *KCNN2* (SK2, K_Ca_2.2), and *KCNN3* (SK3, K_Ca_2.3) genes were detected. We cannot exclude whether these channels are involved in purinergic inhibitory junction potentials since we did not test the responses by electrical field stimulation.

The functional expression of K_ATP_ channels can be tested by K_ATP_ channel openers (lemakalin, pinacidil, cromakalim, NCD, etc.) and the K_ATP_ channel blocker GBC (19, 23). GBC depolarized murine colonic smooth muscle, suggesting that K_ATP_ was basally activated and involved in setting RMP (16). We found that NCD induced hyperpolarization and GBC blocked these effects. However, GBC itself did not cause depolarization. Thus K_ATP_ is functionally expressed but does not regulate the RMP, suggesting that K_ATP_ is not basally activated. *KCNJ8* (Kir6.1) and *KCNJ11* (Kir6.2) are pore-forming subunits of K_ATP_ channels (13, 16). RT-PCR detects only Kir 6.2 transcript in HGCS. We did not examine the expression of sulfonylurea receptors (SUR1 and SUR2B) for this study.

#### Role of Kir and HERG channels in HGCS.

Kir conductances have a property of outward current around K^+^ equilibrium potential (*E*_K_) and thus expression of this conductance contributes in regulating RMP (19). Low concentration of Ba^2+^ (μM range) has been used to investigate the functional expression of Kir channels (19). In a previous report, Ba^2+^ (1–100 μM) depolarized cells along the submucosal surface of the circular muscle layer in canine colonic muscles (6). When the submucosal and myenteric pacemaker regions were surgically removed, higher concentrations of Ba^2+^ were required to depolarize circular muscle. These data suggested that a higher current density of Ba^2+^-sensitive Kir conductance was expressed in ICC but not in smooth muscle cells (6). In the present study, Ba^2+^ (50–500 μM) significantly depolarized HGCS. Patch-clamp data in smooth muscle cells revealed negligible effect of Ba^2+^. RT-PCR showed expression of *KCNJ2* (Kir2.1) mRNA in HGCS. These data support that ICC may express Ba^2+^-sensitive Kir2.1 channels, which are involved in setting the RMP. The importance of a Ba^2+^-sensitive conductance in human gastric electrophysiology has not been recognized previously. We believe this is mechanistic insight into how electrical rhythmicity powers (phasic) peristaltic contractions in the human stomach. If the Ba^2+^-sensitive conductance is not available, depolarization occurs, slow waves are blocked or greatly reduced in amplitude, there is no mechanism for slow wave propagation, and there is no repolarization period between the remaining small slow waves for relaxation to occur. Contraction, in the absence of the Ba^2+^-sensitive conductance, would tend not to be propagating and peristaltic in nature, thus not productive for gastric emptying.

Interestingly, *KCNJ3* (Kir3.1) and *KCNJ6* (Kir3.2) transcripts [G protein-gated inwardly rectifying K^+^ channels, (GIRK)] were also detected in canine colonic smooth muscle and expression of both proteins was confirmed by immunohistochemistry (2). However, the functional role of GIRK channels has not been demonstrated in gastrointestinal tissues. We believe that the transcriptional expression of GIRK may be expressed in neuron or interstitial cells. The functional expression of *KCNH2* (HERG) in the human jejunum has been reported (5). In human jejunum, a low concentration of E-4031 (10 nM) increased the number of spikes per slow wave and a high concentration (1 μM) induced depolarization. In the present study, we did not find a significant change of the RMP (up to 1 μM of E-4031 and 3 μM of ketoconazole) in HGCS. These data suggest that the transcriptional expression of *KCNH2* is not expressed in the muscle layer even though *KCNH2* transcript was detected in HGCS tissue.

In conclusion, Kir and Kv channels are main K^+^ conductances to set RMP in HGCS. Development of isolation technique of ICC and PDGFRα^+^ cell in HGCS is required to confirm molecular and functional expression of K^+^ conductances at the cellular level.

## GRANTS

This study was supported by Samsung Biomedical Research Institute Grant C-B1-125.

## DISCLOSURES

No conflicts of interest, financial or otherwise, are declared by the author(s).

## AUTHOR CONTRIBUTIONS

J.Y.L. and P.-L.R. conception and design of research; J.Y.L., E.-J.K., K.D.A., and S.K. performed experiments; J.Y.L. and P.-L.R. analyzed data; J.Y.L., E.-J.K., and P.-L.R. interpreted results of experiments; J.Y.L. prepared figures; J.Y.L., E.-J.K., and P.-L.R. drafted manuscript; J.Y.L. and P.-L.R. edited and revised manuscript; S.K. and P.-L.R. approved final version of manuscript.
